# University of Pennsylvania

**Published:** 1843-02-18

**Authors:** Henry Sargeant


### UNIVERSITY OF PENNSYLVANIA.

### CLINIC OF DR. W. POYNTELL JOHNSTON.

*At the Locust Street Dispensary, Jan.* 21, 1843.

[Reported from notes by Henry Sargeant.]

### LECTURE II.

Case I.—*Granular blepharitis following measles—application of solid Sulphate of Copper.*—This little girl, æt. 12, was attacked with measles when 7 years old. The measles was accompanied with ophthalmia, and the eyes have remained sore ever since; notwithstanding various collyria which have been employed for their relief. On everting the eyelids, you will perceive that the conjunctiva, of a uniform velvety redness, and slightly puffed, presents a vast number of very minute facets. It exhibits perfectly the granular condition, to which we have already referred, as the result of long continued irritation. This condition serves to confirm what we have previously stated, that nature is generally unable to effect a cure when this condition of the mucous membrane is once established. The disease, in the present instance, has continued unimproved for five years. In this case we have seen that nothing short of an application capable of entirely modifying the granular surface of the conjunctiva, will afford permanent relief. By rapidly sweeping the surface with a solid stick of the nitrate of silver, or with a crystal of the sulphate of copper, the little ulcers rapidly heal, the irritation disappears, and the conjunctiva is restored to its normal condition and functions.

This case will serve, also, to introduce the next, in which a similar alteration, the result of long continued irritation, occupies the mucous membrane of the urethra, and may probably be relieved by a similar remedy.

Case II.—*Long continued masturbation—granular condition of the mucous membrane of the prostatic portion of the urethra—Nocturnal and diurnal pollutions—Imbecility — Hypochondriasis — Application of the solid Nitrate of Silver.*—J——— H———, upwards of 23 years old, presents, you perceive, the ordinary appearance of a lad of 17 or 18. He is emaciated; entirely devoid of beard, and speaks in the soprano voice of a youth before puberty. His gait is unsteady; his eyes habitually downcast; cannot be brought to regard you without trembling; when addressed, he averts his head, while his colour perpetually varies from palor to a deep blush. The pulse is feeble, variable, and occasionally intermittent; the skin is dry and harsh. He gives the following history of his case; but his memory is so defective, that the latter portions of his statement must be received with some degree of reserve. He remembers perfectly all the events of his childhood up to 11 or 12 years. He is also able to recall, with considerable accuracy, circumstances which took place between his 12th and 18th years; but since this period everything has left a confused impression upon his mind, and the occurrences of the last year are lost to him entirely.

It appears that he was in the enjoyment of excellent health until the age of 10 years; at this period he contracted the habit which has brought him to his present wretched condition. For three years he abandoned himself to excessive masturbation, and then had the courage to relinquish the habit for nearly a year, during which his health, which had suffered much, was manifestly improved. At the age of 14 he was apprenticed to a printer in this city, when he again renewed the habit to the same excess as before, (three or four times daily,) and has continued it unremittingly till within the last few weeks. His health, under the influence of this vice, again became impaired; his appetite was capricious; he suffered continually from nausea and headache. He almost invariably rejected his breakfast, and frequently his other meals. He was very often compelled to relinquish work early in the day, on account of the nausea and feeling of general distress from which he suffered. He gradually withdrew from the companionship of his fellows, and sought solitude. He became averse to the amusements of his age; and, although aware that his feelings of utter wretchedness were attributable to the vice into which he had fallen, he was unable to relinquish it. He made the effort, and discovered that if he abstained from masturbation, he was subjected to nocturnal pollutions, (two or three in the course of a night.) At the age of 18, these pollutions first became diurnal, and occurred as frequently as three or four times a day while he was engaged at his work as compositor. These were in turn followed by imperfect erection, and finally by impotence. It is from the period of the diurnal emissions that he dates the first effects upon his intellect. Memory, especially of names and dates, became confused, and, finally, almost entirely lost. He suffered continually from mental anxiety and depression; became remarkably timid; was afraid to retire to bed, and in the dark was tortured incessantly with the horrors, (we use his own expression;) his sleep was troubled and unrefreshing; in the morning he was unwilling to rise, owing to lassitude, and a dull dragging pain in the back.

At the age of 21 he was released from his apprenticeship, but has never since then had sufficient energy to keep employed. After working for a few days, harassed by the most gloomy and depressing images, he shuts himself up in his chamber, where, he assures us, he prays for death as a release from his mental sufferings. He has now relinquished the habit to a certain extent, but still is subject to diurnal emissions, which he cannot control, and which keep him in a condition of most profound melancholy. The manly character in his case cannot be said to have been lost, it has never been developed. He is startled and trembles at the slightest noise; his intellect is now so impaired that he cannot recall the events of yesterday; he is perpetually tortured by frightful images, from which he cannot divert his mind; and while he is conscious that life and intellect are wasting away with the seminal discharges, he is entirely unable to arrest them, and despairs of relief.

This patient, gentlemen, presents a series of symptoms which are by no means rare. They will be frequently found to exist, to a greater or less degree, in young and middle aged men, although the instances are few in which they become so aggravated as in the case before you. These symptoms are not always referred to their proper cause, viz., too frequent seminal emissions, produced by continued excitement of the genital apparatus. The most obvious of the symptoms is mental depression; and the patient, regarded as a hypochondriac, is ordered tonics, baths, and a change of scene, which experience shows to be incompetent to cure. In very many of these cases of hypochondriasis which yield to no treatment, a careful investigation will discover that the genital functions are impaired or lost; and it is to the relief of these that we must address our remedies. The almost constant benefit which results from a treatment thus directed, will justify us in studying as largely as the limit of a clinic will admit, the causes of these emissions; for the emissions may be said to constitute the disease; although an effect themselves, they are the origin and cause of all the other phenomena, mental as well as physical.

It was not intended by nature that the seminal fluid should be discharged as rapidly as formed; it is necessary that a certain excess should remain, to be taken into the system, to circulate with the fluids, in order to give tone and character to the man. Remove the imperfectly elaborated semen prior to the age of puberty by artificial means, and let the habit be continued, and the changes in the organization and character of the individual, which should occur at this period, will not take place; there will be an arrest of development, and, as in the case before you, although the *age* of manhood may be passed, its *character* will never be attained. Still further; should the age of puberty have been passed prior to indulgence in this destructive habit—should the individual be in possession of the intellect, courage, and physical vigor, which are the attributes of this period, they will exist only to be lost. The appearance of involuntary emissions will be the signal for a retrograde movement towards timidity and imbecility, and the sufferer will again become—to use his own almost constant expression—a perfect child.

Masturbation to excess is the most general exciting cause of involuntary seminal discharges—but probably not the only one. Frequent excitement, the result of a lascivious imagination, absolute continence in individuals endowed with strong animal passions, appear occasionally to produce them. We have not unfrequently seen cases where we could refer them to no other source—where masturbation was positively denied, and the denial confirmed by the moral character of the sufferer. But whatever the exciting cause, the effects of involuntary pollutions are always analogous to those enumerated by the patient before you. These effects vary in intensity with the frequency of the emissions, but always present the same type, and can rarely, if ever, we apprehend, be referred to any other source.

We can only at present briefly allude to these effects, the limit of a clinic does not afford us space to enter into details, and they have nearly all been enumerated in their most common order of succession by the patient presented to you. He was first made aware of the injury he was inflicting upon himself, by the loss of energy, and the mental anxiety which he experienced; he was constantly tormented by vague apprehensions, which finally amounted to "horrors," and at present, although he habitually seeks solitude, he fears for an instant to be left alone. His memory next became impaired, he gradually discovered a difficulty in recalling names and dates, and following this all the functions of the brain became, in turn, more and more sluggish, until, as you now perceive, they can scarcely be roused into momentary action; he has almost reached a state of dementia, which is the ordinary termination of this malady, if it be not arrested in time. The records of every insane asylum will exhibit to you a startling proportion of its inmates who have been reduced to imbecility by this cause alone; and your own observations will show you, that many cases of mental derangement are probably rendered permanent by a persistence in the habit of masturbation, which has become uncontrollable. Insanity from this cause constantly assumes the form of dementia, never of mania.

Insanity does not, however, occur abruptly—it is slowly reached by successive stages—a pervading idea of the most depressing character first occupies the mind, the sufferer conceives that he is a species of outcast, hopelessly dying; this leads to a discontented, misanthropic disposition, and subsequently to a profound melancholy, and this latter assumes the character of suicidal monomania; he constantly speaks of death as a release from his sufferings, and appears to desire it, yet we know of no case in which an individual thus afflicted, has possessed the courage—if courage it may be called—to terminate his own existence; on the contrary, while from his expressions he would seem to court death, you will find that constant apprehension of death constitutes his chief source of misery.

The digestive functions are early impaired, the patient is habitually constipated; the constipation does not always depend upon imperfect digestion alone, but is maintained also, as we shall see, by a mechanical obstacle to defecation, an enlarged prostate gland; the appetite from these causes is rendered capricious, and the bowels are habitually distended by gas.

Nervous symptoms are always present in some form, but their form varies in each case. The patient complains ordinarily of palpitation of the heart, of fulness of the head, vertigo and giddiness, of temporary amaurosis, and his gait is frequently staggering, owing to inability to regulate the movements of the limbs. We have seen cases where, in connexion with spermatorrhœa, there existed violent neuralgic pains in the intercostal nerves, in those of the face, and of the lower extremities. In one case a cataleptic condition of the right arm was of frequent occurrence; in another epileptiform convulsions of the chest and diaphragm, without loss of consciousness; in a word, the nervous symptoms, as in hysteria, may assume any form, and simulate various organic diseases.

We have here, then, a succession of general and local symptoms which are obviously connected with the frequent seminal discharges. They are always present when involuntary emissions exist. They vary in intensity with the frequency of the emissions; and we shall find that they subside and even disappear when the emissions are checked. Now we have seen that these emissions are generally caused by excessive masturbation, and are ordinarily found in young and middle aged persons. We do not recall a single instance out of a very large number which it has been our lot to observe, in which the disease

*commenced* after the age of twenty-five, but when once established it may continue for an indefinite period, with or without a persistance in the habit which first produced it.

This appears singular; an individual relinquishes the habit of masturbation entirely, yet the emissions continue involuntarily; the sufferer cannot arrest them, although apprised of the moment at which they are about to occur. The slightest excitement, mental or physical, will produce them, without a sensation of pleasure, and without erection. We have known them to be caused daily by the mere titillation of a razor in the act of shaving. They are not disposed to diminish in frequency, but, on the contrary, to increase; and occasionally, notwithstanding every precaution, and the entire cessation of the habit to which they owed their origin, they terminate in a real spermatorrhœa—an incessant dribbling of imperfectly elaborated semen, mixed with prostatic mucus.

Why is it, that when the cause of the excitement is removed, the effects do not always cease? It is because a long continued irritation has produced an organic alteration in the prostatic portion of the urethra in the vicinity of the ejaculatory ducts and mucous lacunæ, and this organic alteration, once established, becomes a permanent source of irritation to the whole genital apparatus.

The character of this alteration may be inferred from the changes which we know to occur in the palpebral conjunctiva, from a similar cause, viz.: protracted irritation.

Mucous membranes are not all identical—but between the palpebral conjunctiva and the lining membrane of the urethra, there is perfect identity—identity of structure, identity of secretion, identity of diseases which are mutually transferable, and even susceptible of a true metastasis from one to the other. We may then rationally infer that the terminations of these diseases will be identical, and that a plan of treatment which is found effectual to cure in the one, will be equally serviceable in the other.

Now, the result of long continued irritation of the palpebral conjunctiva is the production of a granular condition of its surface; this granular condition is rarely removed by the unaided efforts of nature, but may continue for an indefinite period, and during its continuance is a permanent source of irritation to the whole occular apparatus. Of these facts we have furnished to you abundant proof.

By parity of reasoning a protracted irritation of any portion of the urethra, should be followed by a similar change, and the continued excitement of the prostatic mucous membrane, the effect of masturbation, should result in a granular condition, which once established would persist, and be the centre of permanent irritation, which would radiate through a continuous membrane to all parts of the genital apparatus. Now, this granular condition of the prostatic mucous membrane does really exist in spermatorrhœa. Of this you can readily convince yourselves by examining subjects who have died in our prisons—and who during their solitary confinement generally fall into the habit of masturbation. Addiction to this vice is probably one of the causes of the frequent mental derangement, with which the system of solitary confinement is reproached.

Admitting then, what conforms with reason and is proved by observation—that a granular condition of the prostatic mucous membrane is established—we readily account for all the local symptoms which ensue, and for their persistence.

The irritation is transmitted through the ejaculatory ducts to the *vesiculæ seminales*—causing spasmodic contractions of these reservoirs. The first effect of the spasmodic contraction is to produce too prompt an ejaculation during coition, and in succession to render coition imperfect, and then impossible. The emissions from this cause then become involuntary, at first nocturnal under the influence of a lascivious dream, they are gradually repeated at shorter intervals, under a continually augmented exaltation of sensibility, until they occur without pleasurable sensations, or without erection. Diurnal pollutions next follow, and finally *spermatorrhea.* The fluid is rejected by the seminal vesicles as rapidly as it is received.

Again: the irritation is transmitted through the *vas deferens* to the *testicles.* A greater amount of fluid is secreted by these organs and thrown into the seminal vesicles; but this fluid no longer possesses the qualities of healthy semen; it is imperfectly elaborated, very liquid, and only serves to increase the number and amount of the emissions, while it loses its power of impregnating. This condition of the seminal fluid contributes, of course, with the inability to erect the penis, to produce impotence.

Again: the irritation is transmitted by continuity of tissue to the *bladder.* The urine is almost constantly turbid, and not unfrequently fetid. This condition of the urine is produced partly by the extension of the irritation to the kidneys, modifying their secretion, and causing pain in the loins, and partly by an increased mucous secretion from the internal surface of the bladder itself. Careful microscopical examination has shown that it does not depend upon an admixture of seminal fluid with the urine, as was at first supposed. When spermatic animalculæ are found in the urine. they are found only in the first portion discharged; in the washings of the *urethra* after an emission, the last portions, which are the most turbid, contain only mucus and saline particles.

Again: the irritation extends to the *parenchyma of the prostate gland;* the secretion of prostatic mucus is augmented, and the size of the gland is generally increased. We have seen this gland, in a case which was also examined by Professor Jackson, so much enlarged that the finger could with difficulty be passed between it and the sacrum. The enlargement of the prostate offers a mechanical obstacle to defecation, and thus contributes to the habitual constipation under which the patient labours. The hardened fæces in turn react upon the prostate, and compress it during their passage. Hence a common symptom in this disease. At each effort at stool the compression of the gland forces out a portion of prostatic mucus, of which there is a hypersecretion, and the patient is disheartened, under the impression that it is a seminal emission. The microscope has shown us that the fluid thus discharged is simply prostatic mucus.

Now, to recapitulate: a long continued irritation of the mucous membrane of the urethra, maintained by venereal excitement, confined to that portion of the canal which may be considered as most intimately connected with and forming part of the genital apparatus, terminates in a granular condition of the mucous surface. The granulations constitute a permanent centre of irritation; grouped around the verumontanum, at the orifices of the ejaculatory ducts and of the lacunæ of the prostate gland, extending from their situation to the neck of the bladder, they radiate their influence to all the organs with which they are connected by continuity of tissue, and their existence abundantly accounts for all the local phenomena which we have enumerated.

There remains but one other point of study in the present case. We have referred the general prostration of the patient to the frequent involuntary emissions with which he is afflicted, and we see that these emissions depend probably upon a change of structure in the urethra, which is disposed to persist, an alteration of structure which nature unaided is incompetent to cure. This is the origin, the material cause of all the phenomena, and upon its removal will of course depend their disappearance. Is it possible to restore the prostatic mucous membrane to its normal condition, to allay its sensibility, and prevent it from radiating an irritation throughout the genital apparatus? If so, the emissions should cease when their cause is removed.

Let us again revert to the granular condition of the conjunctiva, remaining for years without improvement, while subjected to no treatment, or to an inefficient one. You have seen these granulations in every instance removed without difficulty by the application of sulph. of copper, or nitrate of silver, in the solid form, and with their removal the irritability of the eye has promptly disappeared. We might infer from this that a similar effect would be produced by a similar application to the prostatic portion of the urethra, and experience proves that such is the case. The solid nitrate of silver applied by means of the instrument of Lallemand, so as rapidly but completely to sweep the whole irritated surface, to coat the granulations with a delicate pellicle, and modify the mucous surface, care being taken not to produce a loss of substance—*not to cauterize*—will be followed by a more or less copious discharge of blood, after each passage of urine. The bleeding will cease in the course of a few days, and already the sensibility of the prostate will be found allayed, and the emissions less frequent; but the effect of the application does not cease here; the parts are stimulated to a more healthy action, and continue to return more and more to their natural condition. On this account, the application should not be repeated at too early a period; nor until full benefit has been derived from the first operation. A return of the emissions is the proper signal for its repetition, which may be required two, three, or four times at longer intervals, before the parts are entirely restored to their normal condition and functions. As the emissions under this plan of treatment diminish in frequency, and finally cease, the melancholy condition of the patient is proportionally improved. The first marked effect is a restoration to hope. From despairing of relief, he frequently becomes sanguine of cure, and this hope is maintained by the successive mitigation of all the symptoms. Time will not permit us on the present occasion to follow the gradual progress of the cure, nor to prove to you that the dangers with which this simple operation has been charged, are entirely imaginary. It has been our lot, during the last six or seven years, to perform it some hundreds of times, and in no single instance has it been followed by an alarming symptom.

Some other points connected with the cure of this affection, we are compelled at present to neglect. We cannot, however, dismiss the subject without referring to the researches of Professor Lallemand upon seminal emissions. It is to him that we are entirely indebted for the mode of treatment which we here exhibit, and if we have not before alluded to the labours of this gentleman, it is because his name is so identified with the subject that incessant reference would have been required throughout the lecture. We apply the nitrate of silver, gentlemen, and will again bring the case before you, in order that you may follow its progress.

